# Microglial Piezo1 mechanosensitive channel as a therapeutic target in Alzheimer’s disease

**DOI:** 10.3389/fncel.2024.1423410

**Published:** 2024-06-18

**Authors:** Erol D. Ikiz, Erin R. Hascup, Chilman Bae, Kevin N. Hascup

**Affiliations:** ^1^Department of Chemistry, School of Integrated Sciences, Sustainability, and Public Health, College of Health, Science, and Technology, University of Illinois at Springfield, Springfield, IL, United States; ^2^Department of Neurology, Dale and Deborah Smith Center for Alzheimer’s Research and Treatment, Neuroscience Institute, Southern Illinois University School of Medicine, Springfield, IL, United States; ^3^Department of Pharmacology, Southern Illinois University School of Medicine, Springfield, IL, United States; ^4^School of Electrical, Computer, and Biomedical Engineering, Southern Illinois University at Carbondale, Carbondale, IL, United States; ^5^Department of Medical Microbiology, Immunology and Cell Biology, Southern Illinois University School of Medicine, Springfield, IL, United States

**Keywords:** amyloid-beta plaques (Aβ plaque), mechanotransducer channels, neuroinflammation, phagocytosis, durotaxis, fatty acid (composition), immunomodulation, Mediterranean diet

## Abstract

Microglia are the resident macrophages of the central nervous system (CNS) that control brain development, maintain neural environments, respond to injuries, and regulate neuroinflammation. Despite their significant impact on various physiological and pathological processes across mammalian biology, there remains a notable gap in our understanding of how microglia perceive and transmit mechanical signals in both normal and diseased states. Recent studies have revealed that microglia possess the ability to detect changes in the mechanical properties of their environment, such as alterations in stiffness or pressure. These changes may occur during development, aging, or in pathological conditions such as trauma or neurodegenerative diseases. This review will discuss microglial Piezo1 mechanosensitive channels as potential therapeutic targets for Alzheimer’s disease (AD). The structure, function, and modulation of Piezo1 will be discussed, as well as its role in facilitating microglial clearance of misfolded amyloid-β (Aβ) proteins implicated in the pathology of AD.

## Introduction

Alzheimer’s disease (AD) is a degenerative brain condition marked by gradual memory loss, declining cognitive abilities, and alterations in behavior, culminating in significant disruptions to activities of daily living. One of the most prominent hallmarks of AD and other neurodegenerative disorders is the accumulation of misfolded protein aggregates with age (Sadigh-Eteghad et al., 2015). Examples include Aβ plaques and neurofibrillary tangles in AD, α-synuclein in Parkinson’s disease, polyglutamine repeats in Huntington’s disease, superoxide dismutase 1 in amyotrophic lateral sclerosis, TAR DNA-binding protein 43 (TDP-43) in limbic-predominant age-related TDP-43 encephalopathy, and prion diseases (Sweeney et al., 2017). The presence of misfolded protein aggregates across various neurodegenerative disorders underscores the imperative of directing efforts toward targeting and degrading these aberrant protein structures. Since misfolded proteins accumulate into toxic aggregates, it is likely that the innate and adaptive immune systems are involved in their clearance (Ciccocioppo et al., 2020). While immune clearance is involved in numerous organs and neurodegenerative disorders, there is still much to be uncovered about the role of mechanical signals in modulating the immunological response to proteinopathies.

A major component of the immunological response in the CNS involves microglia. Microglia are specialized macrophages of the CNS that sense neuropathological conditions by detecting biophysical deviations from brain homeostasis to clear foreign or abnormal formations of protein aggregates via phagocytosis (Ayata and Schaefer, 2020). One of the most prominent examples of such deviations would be stiff Aβ plaques that are much more rigid than normal brain tissue. Recent studies demonstrate that the mechanosensitive and nonspecific cation channel Piezo1 plays an important role in perceiving cellular mechanical stress and converting this into cellular signals. Within microglia, these cellular signals trigger phagocytosis and notably participate in the removal of Aβ plaques in AD animal models (Jäntti et al., 2022). This insight forms the basis for understanding the mechanisms through which Piezo1 can detect abnormally stiff material in the parenchyma that results in the corresponding signal transduction pathways for initiating the innate immune response. Regulating microglial Piezo1 may be a potential therapeutic strategy that warrants bridging the gap between neuropathology and mechanobiology. In this review, the different pharmacological and nonpharmacological approaches to modulating Piezo1 will be explored in the context of translational AD research.

## Structure, function, and molecular modulators of Piezo1 mechanosensitive ion channels

**FIGURE 1:**
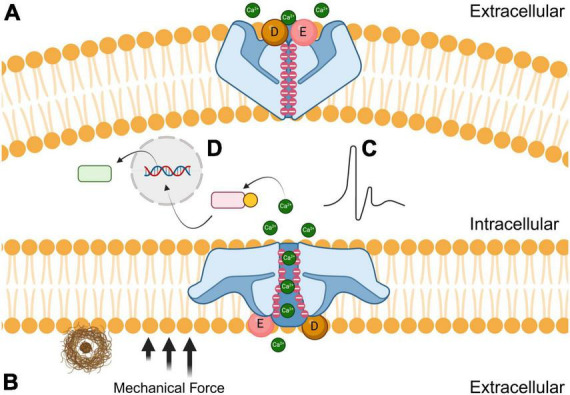
Effects of amyloid plaques (brown) on the closed (**A**; top) and open (**B**; bottom) conformations of Piezo1 receptors. The black arrows represent the mechanical force that induces the open conformation of Piezo1 when microglial encounter stiff amyloid plaques. Negatively charged aspartic (**D**; sphere) and glutamic acid (**E**; sphere) residues are concentrated at the extracellular entrance while negative charges line the channel pore. During mechanical shear stress, cations such as calcium diffuse through the pore resulting in either electrochemical stimulation **(C)** or modulation of signal transduction pathways **(D)** to initiate phagocytosis.

Piezo1 is a trimeric nonspecific cation channel that has an important role in cellular mechanotransduction, in which mechanical stimuli is converted into electrochemical signals (Coste et al., 2010; Xu et al., 2021; Yaganoglu et al., 2023). Piezo1 is localized on many different types of tissues throughout the body, including cardiovascular, musculoskeletal, gastrointestinal, excretory, respiratory, nervous, reproductive, and immune systems (Dienes et al., 2023). Membrane tension and shear stress force these channels from closed to open conformations. Once the force is no longer present, the receptors elastically return to their closed confirmations like a spring recoiling (Lin et al., 2019). Piezo1 has negatively charged aspartic and glutamic acid residues that are concentrated at the cytosolic entrance and throughout the pore, thereby attracting cations and repelling anions (Fang et al., 2021). Human Piezo1 channels are permeable to monovalent cations like lithium, sodium, potassium, and cesium, but are also permeable to most divalent cations that are alkali earth metals like magnesium, calcium, and barium, but not transition metals like manganese (Gnanasambandam et al., 2015). As shown in Figure 1, the opening of Piezo1 by mechanical force allows for the influx of extracellular cations including calcium, which generates a voltage, effectively converting mechanical cues into electrical and chemical signals in cells (Liu et al., 2022). In essence, the mechanosensitive nature of Piezo1 is due to its flexibility and elasticity, while the cation-selective amino acids dictate the voltage transduction. Both the structural and biophysical components are important factors to consider when designing novel therapeutic interventions that can modulate Piezo1 and other mechanosensitive ion channels. There are currently a limited number of biomolecular agonists and antagonists that effectively regulate Piezo1 cation channels despite their ubiquity in different tissues (Romero et al., 2019). It is imperative to understand Piezo1 function to refine the next generation of mechanosensitive ion channel modulators (Tang et al., 2022; Jiang et al., 2023).

Piezo1 has a three-bladed propeller-like structure, with each blade consisting of peripheral transmembrane domains, an anchor that penetrates the inner leaflet of the cell membrane, and a beam that connects the blade to the central pore (Ge et al., 2015; Zhao et al., 2018). The beam is approximately 90 nm long and positioned at a 30-degree angle relative to the plane of the cell’s plasma membrane (Wang and Xiao, 2017). The secondary and tertiary structure of Piezo1’s C-terminal region is responsible for the mechanically modulated physical and chemical properties that determine whether ions permeate through the pore (Coste et al., 2015). Piezo1 also has a unique 38-transmembrane helical structure, and the trimeric propeller-like assembly is thought to curve into the cytosol in its closed conformation (Jiang et al., 2021; Yang et al., 2022).

### Piezo1 channels function independently of each other

As mentioned earlier, Piezo1 is found in a wide variety of physiological organ systems and cell types. While some tissues have a higher density of these ion channels than others, individual Piezo1 receptors appear to function independently of each other suggesting they behave as independent mechanotransducers across the entire cell membrane (Lewis and Grandl, 2021). This finding is important to underscore because it suggests the possibility of activating or inhibiting some Piezo1 channels but not others, which may be useful in different regions of the brain and at different stages of AD progression. Given that microglia preferentially migrate toward stiffer regions, individual Piezo1 or specific clusters of them could be activated and inhibited separately for precise immunomodulation and to avoid systemic inflammation (Bollmann et al., 2015). The key to success for such an endeavor would lie in the development of technologies and systems that can selectively deliver chemical or mechanical modulators to Piezo1 localized on specific cell types or in individual regions of the brain.

### Piezo1 agonists

Yoda1 was the first chemical activator developed for facilitating the open conformation of Piezo1 and is typically given in concentrations ranging between 2 and 20 μM (Syeda et al., 2015). Yoda1 allosterically binds to a pocket approximately 40 Å from the central pore and acts as a molecular wedge that facilitates force-induced conformational changes by lowering the channel’s mechanical activation threshold (Botello-Smith et al., 2019). This mechanism of action underscores the importance of allosteric binding for inducing conformational shifts in Piezo1 channels. More recent research on the activation of Piezo1 by Yoda1 also emphasizes the significance of energetically stabilizing the open conformations of the channel while destabilizing its closed conformations, as well as the dependence of Piezo1 modulation on membrane potential and temperature (Wijerathne et al., 2022). These findings suggest that membrane potential and temperature manipulation as well as the energetic stabilization of desired structural conformations or destabilization of undesired conformations could be additional avenues of research for improving Piezo1 modulation. Yoda2 is a structurally modified version of Yoda1 with the pyrazine ring replaced by 4-benzoic acid. This substitution significantly improves Piezo1 agonist activity with concentrations similar to those of Yoda1 (Endesh et al., 2023). Like Yoda1, Yoda2 also stabilizes open conformations of Piezo1, and its enhanced efficacy is likely due to the negative charge and more desirable pharmacokinetic properties of 4-benzoic acid at physiological pH relative to neutral pyrazine rings (Parsonage et al., 2022).

In addition to Yoda1 and Yoda2, Piezo1 is activated by Jedi1 and Jedi2 (Botello-Smith et al., 2019). Unlike Yoda1, Jedi1/2 activation occurs on the extracellular side of the channel’s three-bladed propeller-like structure (Wang et al., 2018). Furthermore, Jedi1 and Jedi2 do not cross the cell membrane, and therefore only activate Piezo1 from the extracellular side (David et al., 2023). Both Jedi1 and Jedi2 are typically used in concentrations ranging between 5 and 200 μM (Wang et al., 2018). Yaddle1, a recently discovered agonist, modulates Piezo1 channels in concentrations ranging from 0.40 to 1.8 μM. Yaddle1 has a trifluoromethyl group that is thought to wedge itself between the domains of Piezo1, presumably lowering the channel’s mechanical activation threshold and stabilizing its open conformation like the Yoda1/2 agonists (Goon et al., 2024).

### Piezo1 antagonists

In 2000, scientists isolated the GsMT×4 peptide from tarantula spider venom that antagonizes Piezo1 (Suchyna et al., 2000). This peptide has a reversible inhibitory effect on Piezo1 that is independent of voltage (Bae et al., 2011). Other studies involving GsMT×4 have provided some important insights for the current understanding of mechanosensation. For instance, GsMT×4 contains six positively charged lysine residues, effectively repelling cations from mechanosensitive Piezo1 channels (Gnanasambandam et al., 2017). These lysine residues are positioned in the cell membrane immediately surrounding the Piezo1 pore (Suchyna, 2017). This demonstrates the utility of electrostatics in repelling or attracting ions to and from Piezo1 channels. Because GsMT×4 is a peptide from tarantula venom, it can cause substantial pain and can quickly become toxic to the organism. GsMT×4 can therefore only be given in small doses, with concentrations typically ranging from 0.01 to 3 μM (Gnanasambandam et al., 2017).

Ruthenium red, gadolinium, and Dooku1 also antagonize Piezo1, but through varying mechanisms. Ruthenium red and gadolinium are both nonspecific inhibitors of numerous cation channels and they inhibit Piezo1 by blocking calcium transport (Southam et al., 2017; Harraz et al., 2022). Ruthenium red blocks the open conformation on the extracellular side in a voltage-dependent manner while gadolinium binds to phospholipids, thereby compacting the region of the cell membrane surrounding the channel and preventing it from opening (Mulhall et al., 2023; Savadipour et al., 2023). The standard concentrations of both ruthenium red and gadolinium are 10 μM (Vasileva et al., 2021; Yaganoglu et al., 2023). Dooku1 was synthesized by modifying the pyrazine ring of Yoda1, enabling the molecule to attach to the identical binding domain without stabilizing the channel’s open configuration. This modification allows Dooku1 to act as a competitive inhibitor, thereby blocking agonists like Yoda1 from binding to and stabilizing the open conformation of Piezo1 channels (Lacroix et al., 2018). Dooku1 concentrations range between 1 to 10 μM depending on cell type and density (Evans et al., 2018).

## Piezo1 expression and modulating microglial Piezo1 to reduce Aβ plaque burden

### Microglial Piezo1 expression relative to Piezo1 expression in other immune system cells

Piezo1 expression is similar across different types of macrophages throughout the body, including microglia in the CNS, alveolar macrophages in the lungs, Kupffer cells in the liver, and osteoclasts in bones (Tang et al., 2023). Piezo1 is also expressed in immune system cell types including T cells (both cytotoxic CD8s and helper CD4s) as well as memory B cells (Kwak et al., 2023; Karkempetzaki and Ravid, 2024). Piezo1 expression levels in T and B lymphocytes are currently unknown. Piezo1-mediated mechanotransduction activates both T (Liu et al., 2018; Hope et al., 2022) and B cells (Wan et al., 2015; George et al., 2023) via calcium influx. Calcium influx upregulates nuclear factor of activated T cells, a transcription factor that promotes the expression of regulator genes for T cells (Gwack et al., 2007). In B cells, calcium influx activates proinflammatory transcription factors that regulate B cells and promote antibody secretion (Healy et al., 1997). These transcription factors include nuclear factor kappa-light-chain enhancer of activated B cells and c-Jun N-terminal kinase (Dolmetsch et al., 1997). Finally, calcium initiates the differentiation of circulating monocytes into macrophages in various tissues (Murthy et al., 2022) and intercellular calcium signaling potentiates phagocytosis in macrophages (Zumerle et al., 2019).

### Microglial Piezo1 as a stiffness and force sensor in the CNS

Microglia are specialized macrophages of the CNS that are responsible for phagocytosing misfolded proteins and other toxic materials in the extracellular matrices of the brain and spinal cord. Piezo1 can alter microglial phenotypes and activity by sensing differential mechanical stimuli in its environment (Zong et al., 2023). Macrophages, including microglia, tend to migrate toward stiffer and denser mechanical gradient regions in a process called durotaxis (Blaschke et al., 2020). Aβ plaque burden is considered an early pathological hallmark that drives AD progression due to the neurotoxicity of these misfolded protein aggregates (Murphy and LeVine, 2010; Mucke and Selkoe, 2012). Since Aβ plaques are more rigid than the brain parenchyma, microglia migrate toward them (Bolmont et al., 2008; Lewis, 2022). Furthermore, recent studies have demonstrated that Piezo1 senses the abnormal rigidity of Aβ plaques in the CNS via mechanotransduction and that this regulates the microglial clearance of such plaques in AD murine models (Hu et al., 2023). For macrophages to engage in durotaxis, they must be able to detect differences in mechanical gradients, and in microglia, this mechanotransduction is regulated by Piezo1 (Malko et al., 2023). Given the pivotal role of microglia in the innate immunity of the brain, the reduction of microglial Piezo1 expression or impairment of its ability to detect stiffness likely plays a decisive role in the progression of plaque pathology. Considering Aβ plaques continue to accumulate with age and disease progression also indicates that microglial clearance of misfolded protein aggregates gradually fails over time.

### Piezo1’s modulation of microglial migration and proinflammatory cytokine production

Neuroinflammation is one of the most salient and complicated hallmarks of AD and other neurodegenerative disorders and is often mediated by microglia (Subhramanyam et al., 2019). The most recent studies predominantly suggest that microglial Piezo1 expression is typically upregulated during inflammation (Atcha et al., 2021; Yu et al., 2023). Piezo1 channels in microglia regulate the release of proinflammatory cytokines and cell migration in a stiffness-dependent manner (Hong et al., 2023). Moreover, an increase in cell migration was detected in stiff substrates compared to those lacking Piezo1 in knockout microglia (Malko et al., 2023). In rigid extracellular matrices, the upregulation of Piezo1 expression is associated with increased proinflammatory cytokine production by microglia (Zhu et al., 2023). Similar results were also found in astrocytes expressing Piezo1 (Velasco-Estevez et al., 2018). Neurodegenerative disorders typically entail both misfolded proteins and neuroinflammation, so the prospect that Piezo1 addresses both pathological characteristics warrants extensive scientific investigation (Amor et al., 2010; Sweeney et al., 2017). This realization may provide ideas for experiments that augment the current understanding of fundamental mechanophysiology and electrophysiology in the context of biomaterials in the CNS.

### Mechanical gradients, microglial durotaxis, and Piezo1-mediated pro-inflammatory cytokines

The presence of Aβ plaques in the brain is sufficient for the establishment of a mechanical gradient that enables microglial durotaxis. Microglia lacking Piezo1 release fewer proinflammatory cytokines while Yoda1 activation of Piezo1 increases IL-1β, IL-6, and TNF-α secretion (Zhu et al., 2023). Zhu and colleagues also demonstrated that Piezo1 deficiency and the resulting decreased secretion of proinflammatory cytokines enhance microglial migration toward Aβ plaques, indicating that microglia travel down the concentration gradient of such cytokines. While the significance of these results should not be ignored, it is important to underscore that these effects were examined in microglial cell lines and further validation in whole animals is needed. Their findings do suggest that microglial Piezo1 plays a significant role in neuroinflammation initiation and maintenance and that proinflammatory cytokines correlate with reduced microglial migration to Aβ, perhaps highlighting an additional factor contributing to the chronic neuroinflammatory phenotype observed in AD. Microglia are known to phagocytose Aβ plaques and other misfolded protein aggregates (Jäntti et al., 2022). Additional studies are warranted to determine factors influencing microglial durotaxis to enhance the clearance of Aβ plaques.

### The interplay between respiratory infection, microglial Piezo1, and Aβ plaque deposition

Infection induces a systemic inflammatory phenotype to initiate an immunological response and promote healing. Chronic activation of this inflammatory response can have detrimental consequences. Data from experiments conducted in APP/PS1 mice have shown that respiratory infections accelerate disease progression in this AD model (McManus et al., 2014). Moreover, bacterial infection substantially increases the expression of Piezo1 not only in microglia, but also in Aβ plaque-reactive astrocytes and alveolar macrophages located throughout the body (Velasco-Estevez et al., 2018; Geng et al., 2021). It is possible that there is a neurophysiological or immunometabolic threshold that, once exceeded, results in the dysfunction that is seen in dementia and neurodegeneration.

## Nonpharmacological methods for modulating microglial Piezo1 and Aβ plaque clearance

### Disadvantages of using molecules to modulate Piezo1 in microglia and other cell types

The first disadvantage of using molecules to modulate Piezo1 is their inability to cross the blood-brain barrier (BBB). Certain modulators like GsMT×4, are incapable of crossing the BBB due to their polarity (Wang et al., 2016; Suchyna, 2017). Furthermore, GsMT×4 and other ion channel-modulating peptides are derived from venom and cause pain because due to their inherent toxicity (Suchyna et al., 2000). Other Piezo1 antagonists like ruthenium red and gadolinium contain heavy metals that are toxic and can accumulate in adipose tissue (Mabuza et al., 2018; Blythe et al., 2019; Takanezawa et al., 2023). Yoda1 has poor solubility and stability while Dooku1, Jedi1/2, Yoda2, and Yaddle1 are newer modulators and additional studies are needed to determine potential adverse reactions (Wang et al., 2018; Parsonage et al., 2022; Goon et al., 2024). The drawbacks of these molecular modulators have forced researchers to examine nonpharmacological methods to modulate Piezo1 receptors.

### Dietary fatty acids

A rich source of biomodulators in the CNS are fatty acids that constitute the building blocks of cell membranes (Tracey et al., 2018). Variations in lipid bilayer composition can significantly modify membrane mechanics and therefore Piezo1 activity and function (Ivkovic et al., 2022). This is why fatty acids and other lipids modulate Piezo1 activity and its mechanotransduction in microglia for immunomodulation of the brain parenchyma. While these fatty acids may play important roles in the brain, they do not readily cross the BBB, and must therefore be transported via various metabolic mechanisms such as esterification (Pifferi et al., 2021). However, during AD progression, the BBB breaks down, thereby allowing fatty acids to more readily traverse across the microvasculature (Solis et al., 2020). Thus, any therapeutic strategy aiming to utilize fatty acids must take the BBB into account, whether that be a prodrug that can be metabolized into a suitable compound or some other noninvasive or minimally invasive method of delivery.

Recent evidence suggests that microglial metabolism is dynamic and constantly evolving to meet the needs of the brain, thereby altering its pathological and neuroinflammatory responses (Bernier et al., 2020). Given that diet and exercise are known to affect metabolism, modifiable lifestyle changes may mitigate the progression of neuroinflammatory pathologies like Alzheimer’s, Parkinson’s, and Huntington’s diseases. Diet can significantly alter gene expression, and experiments have demonstrated that Piezo1 is elevated in both high-fat diet (HFD) fed male and female mice (Zhao et al., 2019). An HFD also induces metabolic syndrome and chronic inflammation (Duan et al., 2018), which are risk factors for developing AD. Our prior research has shown that a HFD does not affect plaque burden but exacerbates neuroinflammation in the APP/PS1 model of AD (Hascup et al., 2019). Overall, this may provide further explanation as to the brain health benefits associated with a Mediterranean versus Western diet since the primary differences between the two are the types of fatty acids consumed (Simopoulos, 2016).

**FIGURE 2:**
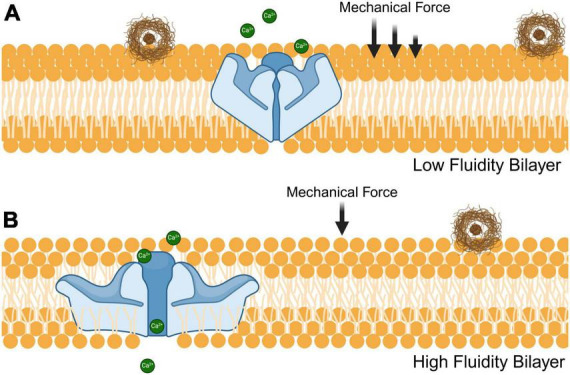
Influence of fatty acid composition on phospholipid bilayer fluidity and Piezo1 receptor sensitivity. The fluidity of phospholipid bilayers is modulated by both unsaturated and saturated fatty acids that have an impact on the ability of Piezo1 to sense mechanical stressors such as stiff amyloid plaques (brown). **(A)** Densely packed saturated fatty acids decrease the membrane fluidity thereby increasing the mechanical stress needed for Piezo1 to achieve an open conformational state. **(B)** Incorporation of unsaturated fatty acids to the phospholipid bilayer reduces its density, resulting in enhanced fluidity and decreased mechanical force required to open the Piezo1 pore.

Dietary fatty acids may also provide a means of therapeutic intervention. The types of lipids consumed can alter membrane stiffness, which directly affects mechanosensitive ion channels (Cordero-Morales and Vásquez, 2018). As shown in Figure 2, saturated fatty acids are densely packed together and therefore increase the stiffness of cell membranes. Thus, more mechanical force is required to induce Piezo1 from a closed to an open conformational state (Romero et al., 2020). This may also explain why Piezo1 is upregulated in rodents that are fed a diet high in saturated fats; as a compensatory mechanism to account for the decrease in membrane fluidity (Zhao et al., 2019). Limiting the intake of saturated fatty acids could reduce the mechanical force needed to activate microglial Piezo1 channels and help the clearance of toxic aggregates of misfolded proteins in the brain. Furthermore, phospholipids containing polyunsaturated fatty acids have been shown to enhance cellular mechanosensation in the CNS (Vásquez et al., 2014). Hence, increasing polyunsaturated fatty acid consumption could improve Piezo1 sensitivity and therefore microglial clearance of protein aggregates. A dietary contrast that holds notable significance concerning fatty acids is the distinction between omega-3 and omega-6. The former is regarded as healthier and linked to the Mediterranean diet, whereas the latter is associated with Western dietary patterns (Simopoulos, 2016). Omega-6 fatty acids, such as linoleic acid and docosahexaenoic acid, cause ion channel dysfunction in both Piezo1 and Piezo2, which is homologous to Piezo1 (Romero et al., 2023). On the other hand, omega-3 fatty acids like eicosapentaenoic acid significantly increases Piezo1 activity (Fatkin et al., 2022). This information can be used to either inactivate Piezo1 with omega-6 fatty acids or activate it with omega-3 fatty acids to control inflammatory responses and microglial clearance.

### Physical exercise

Exercise is a modifiable lifestyle factor and a risk reduction strategy for AD. Considering Piezo1 is ubiquitously expressed throughout various tissues in mammals, exercise positively modulates Piezo1 activity (Dienes et al., 2023). Furthermore, Piezo1 receptors located on endothelial and immunological cells that interact with the BBB are activated because of increased blood flow (Rode et al., 2017). Voluntary physical exercise has been observed to significantly reduce neuroinflammation and enhance the clearance of Aβ plaques (He et al., 2017; Wang et al., 2023). This strongly suggests that physical exercise has therapeutic effects on neuroinflammatory conditions involving the accumulation of misfolded protein aggregates.

### Ultrasound

The biophysical activity of Piezo1 and other mechanosensitive ion channels are stimulated by forces, including ultrasound. Transcranial ultrasound with a frequency of 40 Hz has been shown to activate microglial clearance of Aβ in AD mouse models (Bobola et al., 2020). Given that high-frequency sound waves are a form of mechanical force, and that the conformation of Piezo1 is dependent on the presence or absence of such forces, ultrasound at certain frequencies can induce an open ion-conducting conformation (Ozkan et al., 2023). Ultrasound affects the cell membrane and its Piezo1 in a similar way as sensing stiff protein aggregates. Ultrasound effectively restored microglial activation and reduced plaque burden in both sexes of the 5×FAD amyloidogenic AD mouse model (Bobola et al., 2020). It is important to note that while ultrasound may have affected cell membranes and thereby the Piezo1 channels in it, it is also possible that the frequencies used could be breaking up the plaques. This would allow microglia to phagocytose smaller fragments of Aβ, which would likely facilitate the clearance of plaques from the brain. This procedure is like regular diagnostic ultrasound, which is safe in both humans and non-human primates, and therefore, has immediate translational implications for human AD patients.

### Optical control of Piezo1

Light therapy has been gaining recognition for its importance in health and disease. While electromagnetic radiation may not change the mechanical properties of cell membranes or be a mechanical wave that must travel through a medium, it is effective at transmitting energy. Recent studies have demonstrated that light-induced molecular motion is capable of opening Piezo1 in a manner like that of more traditional mechanical cues (Peralta et al., 2023). It is likely that the initial application of optical control for Piezo1 will be limited to researchers attempting to glean novel insights pertaining to the channel’s structure and function.

## Conclusion

This review discussed various functions of microglial Piezo1 in the context of CNS proteinopathies and its potential role as a therapeutic target to treat AD. Considering that misfolded proteins are the pathological hallmarks of several other neurodegenerative disorders, Piezo1 modulation may also have broader therapeutic potential. The methods of both activating and inhibiting Piezo1 that were mentioned in this review will likely increase in number in the coming years and the ones that were discussed will probably increase in sophistication and range of applications. Such progress will make it an exciting time to study Piezo channels but also mechanobiology and mechanotransduction in various pathological contexts.

## Author contributions

EI: Investigation, Writing – original draft, Writing – review & editing. EH: Writing – original draft, Writing – review & editing. CB: Funding acquisition, Writing – original draft, Writing – review & editing. KH: Conceptualization, Funding acquisition, Supervision, Writing – original draft, Writing – review & editing.
